# Artificial Intelligence (AI) in Rare Diseases: Is the Future Brighter?

**DOI:** 10.3390/genes10120978

**Published:** 2019-11-27

**Authors:** Sandra Brasil, Carlota Pascoal, Rita Francisco, Vanessa dos Reis Ferreira, Paula A. Videira, Gonçalo Valadão

**Affiliations:** 1 Portuguese Association for CDG, 2820-381 Lisboa, Portugal; sd.brasil@fct.unl.pt (S.B.); cm.pascoal@campus.fct.unl.pt (C.P.); rab.francisco@campus.fct.unl.pt (R.F.); p.videira@fct.unl.pt (P.A.V.); 2 CDG & Allies—Professionals and Patient Associations International Network (CDG & Allies—PPAIN), Faculdade de Ciências e Tecnologia, Universidade NOVA de Lisboa, 2829-516 Lisboa, Portugal; 3 UCIBIO, Departamento Ciências da Vida, Faculdade de Ciências e Tecnologia, Universidade NOVA de Lisboa, 2829-516 Lisboa, Portugal; 4 Instituto de Telecomunicações, 1049-001 Lisboa, Portugal; goncalovaladao@gmail.com; 5 Departamento de Ciências e Tecnologias, Autónoma Techlab–Universidade Autónoma de Lisboa, 1169-023 Lisboa, Portugal; 6 Electronics, Telecommunications and Computers Engineering Department, Instituto Superior de Engenharia de Lisboa, 1959-007 Lisboa, Portugal

**Keywords:** artificial intelligence, big data, congenital disorders of glycosylation, diagnosis, drug repurposing, machine learning, personalized medicine, rare diseases

## Abstract

The amount of data collected and managed in (bio)medicine is ever-increasing. Thus, there is a need to rapidly and efficiently collect, analyze, and characterize all this information. Artificial intelligence (AI), with an emphasis on deep learning, holds great promise in this area and is already being successfully applied to basic research, diagnosis, drug discovery, and clinical trials. Rare diseases (RDs), which are severely underrepresented in basic and clinical research, can particularly benefit from AI technologies. Of the more than 7000 RDs described worldwide, only 5% have a treatment. The ability of AI technologies to integrate and analyze data from different sources (e.g., multi-omics, patient registries, and so on) can be used to overcome RDs’ challenges (e.g., low diagnostic rates, reduced number of patients, geographical dispersion, and so on). Ultimately, RDs’ AI-mediated knowledge could significantly boost therapy development. Presently, there are AI approaches being used in RDs and this review aims to collect and summarize these advances. A section dedicated to congenital disorders of glycosylation (CDG), a particular group of orphan RDs that can serve as a potential study model for other common diseases and RDs, has also been included.

## 1. Introduction

In Europe, a disease is considered rare when it affects less than 1 in 2000 people [1]. There are more than 7000 rare diseases (RDs) worldwide [2]. About 80% of RDs have a genetic origin and approximately 75% affect children [1,2]. Although individually rare, collectively, RDs are estimated to affect 350 million people globally [3,4].

RDs’ underrepresentation in research and treatment development is related to the following aspects: (a) underestimation of true disease frequency owing to the lack of RDs’ awareness, as well as specific diagnostic criteria and official clinical guidelines [5,6]; (b) lack of funding, which hampers basic and clinical research [6]; (c) small and geographically dispersed patient alongside with the scarcity of specific coding systems, which affect patient recruitment for clinical research leading to underpowered trials [5,7]; (d) insufficient knowledge of the disease pathophysiology and natural history combined with the insufficiency of validated outcomes and disease-specific biomarkers delays the establishment of clinical trials [5]; (e) limited interest of pharmaceutical companies associated with the high cost of drug research and development (R&D) and the small orphan drug market [7].

Despite all of these challenges, the development of new technologies, such as genomic analysis by means of next generation sequencing (NGS) and other “omics technologies” has boosted RDs’ diagnosis and molecular understanding [8,9]. Information retrieved from these technologies represents a substantial increase in data that need to be selected, analyzed, and integrated [9]. This “big data” age constitutes a huge opportunity for the progress of therapy R&D in RDs, but also poses significant data management and ethical challenges.

Artificial intelligence (AI), mainly through machine learning (ML, a subtype of AI), provides algorithms capable of learning from data. According to the U.S. Food and Drug Administration, AI is “the science and engineering of making intelligent machines”, while ML is “an AI tool that can be used to design and train software algorithms to learn from and act on data” [10]. ML algorithms can uncover complex data patterns and can be stowed in one of the following categories: (i) supervised learning (ii), unsupervised learning, or (iii) reinforcement learning [11].

In supervised learning, the data are labeled, that is, the algorithm is given the input data along with the corresponding target data. The algorithm’s task is then to uncover the relation between inputs and corresponding targets. Tasks of classification (e.g., whether or not a chest X-ray corresponds to a tuberculosis patient) [12] and regression (e.g., predicting breast cancer risk from a magnetic resonance image using logistic regression) [13] are examples of supervised learning.

In unsupervised learning, there are input data, but no targets. The algorithm’s job is to unveil some underlying structure in the data. The tasks of clustering, which is the automatic assignment of object groups into clusters (groups) (e.g., study and treatment of Alzheimer’s disease) [14], and density estimation (e.g., analyzing co-morbidities of migraine) [15] are medical examples of unsupervised learning.

Finally, in reinforcement leaning, the algorithm’s aim is to find the most suitable action in order to maximize a reward, which, in turn, depends on the action (e.g., dynamic treatment policies [16] and epilepsy prevention [17]).

In the last decade, these and other deep learning algorithms proved to be effective in many areas, leading frequently to state-of-the-art results.

The use of AI algorithms in diagnosis (e.g., analysis and classification of sequence pathogenic variants) and drug discovery and preclinical research (e.g., new molecules designing, animal testing reduction, drug interaction) are just some of the examples of the benefits AI has brought to biomedicine [18]. Most of these tools are applicable to a wide spectrum of diseases, including RDs. Taking into account all these advances, we aim to understand where we stand as for the use of AI in RDs. We performed a literature revision, having collected and structured information regarding the application of AI in several domains of RDs; namely, (a) diagnosis and prognosis, (b) disease classification and characterization, (c) therapeutic approaches, and (d) patient registries and health records. Moreover, we highlight AI applications in a specific group of RDs—congenital disorders of glycosylation (CDG).

This review compiles AI-based tools for biomedical research and clinical practice, increasing the knowledge and awareness on AI applications in RDs.

## 2. Materials and Methods

For this review, a double and triple combination of keywords related to RDs and CDG, AI (e.g., ML and neuronal networks), and medicine/pharmaceutics was used to search the Medline database, using PubMed as the search engine through its application programming interface (API) Entrez Programming Utilities [19] (Figures S1 and S2). To use that API, we wrote a Python programming language script using libraries from the biopython project [20] (Supplementary Note and Figure S3). The results for each of the keyword combinations were limited to the twenty most relevant papers. Duplicate entries were eliminated and a refinement of the results was performed based on the title, abstract, and MeSH terms of each publication. Three rounds of manuscript selection were performed: (1) articles were selected based on title and abstract reading by four researchers; (2) the ones matching the selection criteria were included for the second round of full-manuscript reading; (3) a final round was performed by two independent researchers, who analyzed all the full-texts, to guarantee uniform selection criteria of all included manuscripts.

Inclusion/exclusion criteria were as follows:

1. Only English-written manuscripts that included title, abstract, and MeSH terms were selected;
2. Manuscripts expressing the usage of concrete AI algorithms (or families of algorithms) to address specific problems related to RDs were included;
3. Orphanet classification was adopted and only RDs with Orpha codes were included;
4. Reviews were excluded from the results, although we have included some for contextualization purposes. References from the included reviews were screened to guarantee that no relevant manuscripts were missed.

The list of available AI- and ML-based methods found in the included articles can be found in Table 1.

A classification according to the learning type (unsupervised and supervised), as well as advantages and disadvantages of the major AI-/ML-based methods described in this review can be found in the Supplementary Materials (Table S1).

## 3. RDs’ Prediction: Diagnosis and Prognosis

### 3.1. Mutation Detection and/or Prediction

The identification of disease-related genes and mutations is essential for diagnosis and disease prediction [21]. NGS, particularly whole-exome sequencing (WES) and whole genome sequencing (WGS), is now firmly established in RDs’ diagnostic and research laboratories [22,23]. The advances made in these technologies make them affordable and indispensable, but the interpretation of the effect of the discovered new variants remains a challenge [22].

#### 3.1.1. Single Nucleotide Variants

The detection and screening of pathogenic amino acid changes from background polymorphisms is imperative for efficient use of NGS in personalized medicine [22,24]. Several tools have been created and used to detect mutations and predict its effects.

Non-synonymous single nucleotide variants (SNVs) that alter protein sequence are strong candidates for disease-causing variants. Carter et al. described the variant effect scoring tool (VEST), a new method that uses random forest (RF) to prioritize missense variants that alter protein activity [21]. VEST was applied to Miller and Freeman Sheldon syndromes’ exomes, placing the causal genes among the top two when only Mendelian genes were considered. The VEST tool is advantageous because allele frequencies are not required and it performs even without controls [21].

CliniPred, an ensemble classifier for predicting disease relevance of missense SNVs, which uses a combination of two different ML algorithms (RF and gradient boosting models—Table 1), was applied to clinical data from 31 different RDs’ exomes, showing a high performance. Furthermore, predictor performance increased with the addition of allele frequency [22]. SNV prediction methods are generally sequence-based only or sequence and structure combined. Structure-based features (e.g., binding site) may act as important determinants for deleteriousness of loci in RDs. However, there has not been an extensive examination of structure-based features for mutation prediction [25]. Weka, a data-mining software that contains multiple ML methods to analyze the combination of a RDs’ rare variant dataset (ClinVarRVRD) and protein structures from Protein Data Base, has shown that the combination of structure- and sequence-based features improves rare variant analysis [25]. Synonymous SNVs may contribute to RDs (e.g., by changing the splicing pattern, as well as protein fold). However, most pipelines for the identification of disease-causing mutations filter out synonymous SNVs at the early stages, concentrating on amino acid altering and regulatory variation [26]. Buske et al. have developed the silent variant analyzer (SilVA), an RF-based prioritization method for synonymous variants in the human genome. SilVA considers multiple features based on sequence conservation, splice factor motifs, splice donor/acceptor sites, RNA folding energy, codon usage, and CpG content. SilVA was used to analyze seven synonymous variants found in Meckel syndrome families and 12 synonymous variants discovered in a group of other RDs. Despite the small number of training examples, SilVA is a useful method to prioritize synonymous SNVs [26]. A set of supervised learning tools was used to compare and analyze the effect of variants upon the structure, function, and pathology of the Mevalonate kinase protein (ORPHA:309025). Three groups (mild, intermediate, and severe) were obtained, although the discrimination between groups was not sufficient to make meaningful predictions about variants of unknown severity [27].

A support vector machine (SVM) algorithm was used to predict single nucleotide polymorphisms (SNPs) significance in common variable immunodeficiency (CVID). Three hundred and sixty-three CVID patients were genotyped with 610,000 SNPs, with 1000 being strongly predictive of CVID [28].

The analysis of non-and synonymous SNVs using different AI algorithms has proven to be fundamental for the diagnosis of RDs.

#### 3.1.2. Slicing and Multigenic Mutations

Noncoding regions account for 90% of causal disease loci in genome-wide association studies of human complex diseases, suggesting that penetrant noncoding variants may be the cause of mutations in RDs [29]. SpliceAI, a deep residual neural network (NN), predicts the presence and absence of splice donors and acceptors in pre-mRNA transcripts. It was used to assess the splice-altering impact of a pathogenic cryptic splice variant in the MYBPC3 intron associated with hypertrophic cardiomyopathy (ORPHA:217569) [29]. The discovery and analysis of cryptic splice mutations represents a new diagnostic and therapeutic avenue in RDs.

In RDs, the classic concept of one gene leading to a particular phenotype has been shown to be an oversimplification. Oligogenic or multilocus genetic patterns have been discovered for diseases initially considered to be monogenic. Hence, the variant combinations pathogenicity predictor (VarCoPP), an ML approach to identify pathogenic variant combinations in gene pairs, was developed by Papadimitriou et al. [30]. VarCoPP was trained using combinations involved in known oligogenic diseases providing robust 95% and 99% confidence labels. Thus, VarCoPP is a clinically important tool, making more informative and accurate prediction than those based solely on monogenic variant pathogenicity scores [30].

#### 3.1.3. Copy Number Variation Analysis

Human copy number variants (CNVs) can cause disease by gene dosage, disruption, g fusion, or position effects; hence the analysis of CNVs has become routine in genetic diagnosis. Several online databases and web search services for the analysis of CNVs have been created; however, there is still the need to search and read the original literature for rare CNVs. Yang et al. employed a state-of-the-art literature mining method—using DNorm, an ML powered tool that performs named entities recognition (NER)—to generate CNVdigest, a web-based system containing 1582 CNVs and 2425 diseases. CNVdigest performs and identifies CNVs-disease associations, and has shown interesting results in DiGeorge Syndrome (ORPHA:567) [31].

#### 3.1.4. Genotype–Phenotype Integration

An RF model (Table 1) was used to develope eDiVA (exome disease variant analysis), a variant pathogenicity prediction and annotation tool, which has an innovative pathogenicity classifier (eDiVA-Score). eDiVA was applied to several RDs (e.g., cystic fibrosis and phenylketonuria, among others) and identified known causal disease variants with high precision and recall. Interestingly, prioritization of candidate variants can be enhanced by the addition of clinical information [23].

Both Xrare (using gradient boosting decision tree) and deep phenomeNET variant predictor (DeepPVP, a deep NN-based tool) prioritize disease-causing variants based on a set of phenotypic and genetic features. Xrare and DeepPVP outdid other analogous tools (e.g., CADD and Exomiser) and clinical-expert pipelines at variant prediction [32], even at identifying genes previously unlinked to a disease [33]. These results suggest that these AI methods may be particularly useful to study RDs and rare variants [32,33].

In summary, AI and ML methods can efficiently predict disease-causing genes and mutation types across variable sets of RDs and accounting for different auxiliary measures (e.g., phenotype), thus accelerating and improving diagnosis accuracy.

### 3.2. Phenotype and Biochemical Fingerprinting-Driven Diagnosis

Although medicine has entered the genomic era with NGS, the molecular cause of many diseases is often hard to determine. Hence, clinical phenotype is an invaluable resource for accurate and faster diagnosis of RDs [4]. Electronic health records (EHR) and biomedical literature contain extensive clinical information with huge potential in the development of decision support systems (DSS).

#### 3.2.1. Phenotype-Driven Diagnosis

The heterogeneous association network for rare diseases (HANRD) is a phenotype-driven RD gene prioritization system. It is a network of ontological and curated associations between phenotypes, diseases, genes and pathways obtained using an information propagation algorithm—graph convolution-based association scoring—which infers new binary associations between the terms in the initial network. This tool is a significant advance in diagnosis DSS (DDSS), as it showed better performance compared with other state-of-the-art tools (Orphamizer) [34]. Paul et al. combined rule mining and the Dempster–Shafer theory to calculate probabilistic associations between sets of clinical features and six skeletal dysplasia types recorded in the European Skeletal Dysplasia Network patient registry. Despite data sparseness, the mixed algorithm outperformed specialist medical diagnosis and five different ML methods [35]. For Mucopolysaccharidosis type II (MPS II, ORPHA:580) diagnosis prediction, a naïve Bayes classifier was developed and trained with literature data. The application of this classifier to an unstructured EHR with 505,526 patients classified 125 as possibly having MPS II with 99% accuracy and 84% sensitivity [36]. Additionally, a DDSS based on phenotypic information extraction using collaborative filtering in combination with natural language processing (NLP) was created. Firstly, it was applied to an EHR database encompassing multiple RDs, afterwards being improved by the addition of literature mining [37,38]. Ada XD is a promising DDSS prototype using the augmented Quick Medical Reference (QMR) Bayesian network with an incorporated probabilistic reasoning engine. Ada XD RD patients’ dataset derives from manually annotated and continuously updated EHR and medical literature data. In contrast to the aforementioned DDSS, Ada XD does not require a unique/specific nomenclature (e.g., HPO) [4].

Phen–Gen is a method that uses a systematic Bayesian framework to combine patients’ sequencing data and symptoms from distinct sources. It has been applied to RDs with highly promising results in both coding and non-coding variants. It also outranked the accuracy of genotype-only approaches at variant prediction by 52% [39].

Clinical data warehouses (CDWs) are built on top of EHRs and enable the collection and secondary use of healthcare data. Dr. Warehouse is an open-source CDW based on a vector space model, which explores NLP using the Unified Medical Language System Metathesaurus^®^ (UMLS^®^) [40]. By leveraging phenotype similarities identified in the EHR, Dr. Warehouse can accelerate RDs’ diagnosis and clinical research recruitment [41].

In summary, the integration of patient information has proven fundamental to accelerating diagnosis.

#### 3.2.2. Imaging-Based DDSS

Many RD patients present characteristic dysmorphology [42]. Images are one of the types of data that AI, namely deep learning, is more fruitful at analyzing; hence, imaging is crucial in phenotyping, diagnosis, and even the identification of new RDs [43].

DeepGestalt is a deep learning facial analysis framework for genetic syndromes’ classification that powers a phenotyping platform named Face2Gene [44]. A crucial feature of deep learning is to discover abstract representations of the data (in this case, facial images) that highlight attributes that enable effective learning [45]. The authors use transfer learning to translate the general face recognition learning trained on a large scale dataset to the rare genetic syndromes’ domain. They conclude that DeepGestalt can be used to suggest a diagnostic direction in clinical practice. Several studies have employed DeepGestalt to diagnose rare syndromes with a moderate [46] to high degree of success, namely, Cornelia de Lange syndrome (ORPHA: 199) [47], Emanuel syndrome (ORPHA:96170), and Pallister–Killian syndrome (ORPHA: 884) [48]. Besides diagnosis, DeepGestalt can also be applied to discover new RDs [49].

Other algorithms applied to facial images and neuroimaging have been used to diagnose RDs. For instance, SVMs have been used with facial images for acromegaly (ORPHA:963) [50] and with neuroimaging for the prediction of Pick disease (ORPHA:282) and amyotrophic lateral sclerosis (ALS, ORPHA: 803) presenting with primary progressive aphasia [51].

Hsieh et al. have integrated phenotypic information—generated from AI-based literature and EHR text-mining and/or from facial imaging analysis—to assist in genetic variant interpretation. This ingenious approach (called prioritization of exome data by image analysis—PEDIA) was carried out in a large cohort of patients with monogenetic RDs. The authors show that the addition of phenotypic data significantly improved correct disease-causing gene prediction, particularly when deep learning face recognition (with Face2Gene) was encompassed [52].

#### 3.2.3. Biochemical Fingerprinting

Despite the increase of genetic diagnosis in RDs, for several diseases such as hereditary hemorrhagic telangiectasia (HHT, ORPHA:774), genetic testing is a costly and inefficient option. Lux et al. describe the use of infrared (IR) spectroscopy allied to artificial NN for HHT diagnosis. Two independent studies showed the existence of an HHT-specific IR fingerprint. Hence, IR spectroscopy combined with artificial NN can be considered a serious alternative diagnostic method [53].

In summary, AI can integrate one or several stages of DDSS development: from image recognition and mining and annotation of phenotypic terms, to disease ranking and prediction and positively impacting biochemical-driven diagnosis, ascertaining itself as a multifaceted diagnosis enhancer.

### 3.3. Prognostic Markers

A survival prediction deep learning algorithm (including loss of follow-up cases) was applied in synovial sarcoma (SS, ORPHA:3273) patients. This novel algorithm achieved better performance than a conventional NN based on an alive or dead binary classification, as well as that of the Cox proportional hazard model, the gold standard method for survival prediction in cancers [54]. A complex framework of AI algorithms, including t-distributed stochastic neighbor embedding clustering, deep NNs (using principal components analysis (PCA)), RF, k-nearest neighbor, and regulatory networks, was used in soft tissue sarcomas to identify novel diagnostic and prognostic markers, as well as potential therapeutic targets. Furthermore, the application of different ML methods has improved the understanding of rare cancers [55]. These tools can not only predict disease diagnosis/prognosis, but also simulate therapeutic options, thus guiding better individualized treatment.

## 4. Disease Classification and Characterization

### 4.1. Disease Mechanisms

The unraveling of underlying affected genetic and molecular players and pathways is crucial for disease comprehension and therapeutic target selection. However, gene expression anomaly detection and its correlation with molecular mechanisms and clinical phenotypes is a long-standing ML challenge. The characterizing systematic anomalies in expression (CSAX) data, which operate with feature regression and classification, were applied to a set of trisomy 18 (ORPHA:3380) samples. CSAX data identified 10 top genes to be related to tooth development, immune processes, and glucocorticoid metabolism, which is in accordance with the described clinical phenotype [56]. Small datasets derived from basic research in RDs are challenging for ML. To overcome this, Taroni et al. resorted to a publicly available gene expression database (recount2) and trained a pathway-level information extractor matrix factorization framework with multiple diseases (MuliPLIER). When applied to RDs datasets (specifically systemic lupus erythematosus (ORPHA:536), granulomatosis with polyangiitis (ORPHA:900), microscopic polyangiitis (ORPHA:727), and eosinophilic granulomatosis with polyangiitis (ORPHA:183)), MultiPLIER could describe biological pathways and processes more effectively than the models trained with specific disease datasets [57].

Microarray technology has led to great leaps in molecular disease characterization; nonetheless, it concomitantly created several analysis and clinical interpretation challenges caused by, for instance, genomic noise. Biomedical robots are sets of algorithms or classifiers that can analyze data, create new knowledge, and even act as medical DSS. DeAndrés-Galiana et al. built an AI biomedical robot (supervised filter feature selection methods and dimensional reduction algorithms) with the goal of improving microarray analysis sensitivity and knowledge inference. They used this tool in ALS and inclusion body myositis (IBM, ORPHA:183) patients. In the former, three small-signature genes were identified with 96.5% accuracy, whereas in the latter, 12 genes (enriched in HLA-x family genes) were singled out with an accuracy of 97.5%. Additionally, the biomedical robot could distinguish IBM and polymyositis (ORPHA:732) with 100% accuracy. On the basis of these small-signature genes, various signaling pathways were suggested to be involved in the pathophysiology of these RDs [58]. In another attempt to tackle noise in microarray reading, authors used microarray synthetic modeling to compare different gene ranking methods (fold change, Fisher’s ratio, percentile distance, and entropy) and significance analysis of microarrays. They used the aforementioned ALS and IBM datasets and concluded that the Fisher ratio was the most precise method with the highest discriminatory power [59].

Gaucher disease (ORPHA:355) is caused by lysosomal enzyme glucocerebrosidase-1 deficiency. Aggregating molecular dynamics and deep learning (convolutional variational autoencoder), a model of interaction between this protein and its chaperone-saposin C was designed. This model also predicted how disease-causing mutations destabilize this interaction, uncovering a relevant disease mechanism [60]. Exploring the potential of unsupervised deep ML (DeepNEU) to create a cellular disease model, Danter et al. successfully developed in silico induced neuronal pluripotent stem cells for Rett syndrome (ORPHA:778) [61]. This in silico approach is cost-effective and may have tremendous implications in disease pathways and therapeutic target clarification. Different AI and ML methods allow for the study and prediction of disease mechanisms at distinct levels: from gene to protein and cells.

### 4.2. Disease Categorization and Characterization

The lack of data, complexity and heterogeneity within and among RDs, and overlapping and infrequent clinical manifestations challenge correct disease classification. Often, disease classification consists of numerous, sequential, and expensive clinical and laboratorial tests. AI and ML represent solutions to detect and learn low frequency patterns, delivering automated class attribution, which facilitates correct treatment recommendation.

The characteristic feature mining algorithm (CFML) has uncovered the key features of 15 bone dysplasia disorders resorting to the European skeletal dysplasia network. Although this algorithm showed relatively low average precision (26.73%) and recall (24.68%), it still outdid a standard algorithm for class association rule mining. Furthermore, the CFML selected hallmark features were discriminative in 12 of the 15 disorders [53]. More recently, a stochastic gradient boosting narcolepsy type 1 (ORPHA:2073) versus type 2 (ORPHA:83465) classifier was set up using the European narcolepsy network data. It identified 15 influential predictors (99% accuracy, sensitivity, and specificity), with some of them not included in the current narcolepsy classification criteria. Importantly, revisiting the only misclassified case uncovered a previously incorrect clinician-made diagnosis.

An RF model was used to classify acute liver failure (ORPHA: 90062), determine its etiology, and improve diagnosis in the clinic, achieving an 80% accuracy score in the etiology assignment. Bayesian comorbidity networks and subsequent random walk clustering have been used to discriminate and cluster pediatric pulmonary hypertension (PH, ORPHA:182090) subtypes. Furthermore, Bayesian comorbidity networks were capable of distinguishing rare subtypes and comorbidity associations, even suggesting the existence of a previously unidentified subtype with neurological involvement. In patients with juvenile idiopathic arthritis (ORPHA:92), an identical framework stratified patients according to clinical and laboratorial pattern recognition. Probabilistic PCA and Gaussian mixture model clustering using Bayesian information criterion were applied to categorize the meaningful clinical and biological patterns and to cluster patients, respectively. The originated five subpopulation clusters were clinically relevant and strongly predictive of disease evolution. A semantic text-mining approach using PhenomeNET to compute and predict phenotype-disease relations of 6000 diseases was created. Following this, disease–disease similarity cluster networks based on phenotypic features were built employing the parameter-free clustering algorithm FLAME. Interestingly, phenotypic relationships joined several lysosomal storage diseases (LSD) and, in their vicinity, identified two forms of spinal muscular atrophy, a phenotype not commonly associated with LSD. However, a patient with Charcot–Marie–Tooth disease type 4J (ORPHA: 139515) presenting LSD symptomatology was described [62]. Thus, besides improving disease classification, this AI-based methodology may help diagnose rarer disease (sub)types.

A distinct approach towards phenotype elucidation for better disease comprehension entails the use of deep learning methods to clarify the relationship between disease and disability. Deep NNs were applied to a corpus of scientific abstracts manually annotated for disability-related terms in RDs. This methodology not only detected disability-related terms with an 81% F-measure, but also—via unsupervised learning—established a relationship between disabilities and diseases with 75% accuracy.

## 5. Therapeutic Approaches

Currently, only approximately 5% of the RDs have a treatment [8]. AI presents as a potential game-changer in this field, backed up by the encouraging results obtained in treatment discovery in common disorders.

### 5.1. Drug Repositioning

Drug repositioning—a strategy to identify new therapeutic uses for approved or investigational drugs—has been gaining momentum as a quicker, safer, and cheaper approach, particularly in RDs. AI has emerged as a highly attractive tool to boost drug repurposing for RDs. Lee et al. utilized an ML unified computational framework, URSAHD (unveiling RNA sample annotation for human diseases), which integrates genetic and molecular information about thousands of complex disorders, to test drug repurposing. On the basis of hallmark processes and mechanistic similarities among common/treatable and rare/untreatable diseases, URSAHD was able to predict cisplatin as a therapeutic agent to refractory anemia with excess blasts (ORPHA: 86839) and resveratrol as a potential candidate for sideroblastic anemia (ORPHA:1047) treatment [63]. The prediction made by algorithms in both studies must be experimentally validated and, even though there is still much room for improvement, AI can act as a drug discovery enhancer.

### 5.2. Clinical Trials

Clinical trials (CTs) are pivotal for therapy development. Nonetheless, only a small fringe of RDs have CTs owing to (i) difficulty in patient identification and recruitment; (ii) challenges associated with data obtention and analysis in small populations; and (iii) lack of reliable biomarkers, among others [64,65,66]. AI may provide solutions for these obstacles of CTs in RDs.

#### 5.2.1. Patient Recruitment and Identification

The application of four data mining computable phenotype algorithms to EHR identified 413 new pediatric patients with PH. Other major advantages of this methodology are that (i) it allows for continuous patient recruitment and (ii) once validated, it is transferable to other settings [67].

#### 5.2.2. Biomarkers

Reliable biomarkers help in the identification of normal versus pathogenic processes and/or in the assessment of the response to therapeutics or other interventions, being essential for therapy development [68,69]. In an olesoxime trial for ALS, the application of the Biosigner algorithm (using partial least square discriminant analysis, RF, and SVM) to a pharmacometabolic approach discerned differences in the metabolic profiles between the control and treatment arms. It also recognized sphingomyelins as the most relevant disease progression marker [70]. Moreover, in West syndrome (ORPHA:3451) patients, the employment of this algorithm identified serine and an unknown metabolite (X363) as potential disease biomarkers [71]. Hence, the prediction of disease progression from early metabolomic profiles allied to pharmacometabolomics represent a promising avenue for biomarker detection.

Carlier et al. designed an innovative in silico clinical trial to test bone morphogenetic protein treatment in congenital pseudarthrosis of the tibia associated with neurofibromatosis type 1 (ORPHA:636). Ward hierarchical clustering was employed to stratify the virtual subject population in adverse responders, non-responders, responders, and asymptomatic. Additionally, an RF-based algorithm predicted potential biomarkers for therapy effectiveness, namely rate of cartilage formation (Pmc) and Y3cb [72]. All in all, AI-based methods can resolve many challenges associated with clinical trials in RDs, particularly in combination with other expanding fields like systems biology.

## 6. Patient Health Registries and Medical Records

With the exponential growth of patient data in health records and patient registries, more and better NER methods are indispensable. NER is the process of identifying various semantic classes of terms (e.g., genes, proteins, and diseases) in raw text. It is the first step in the process of knowledge discovery and data mining. Nevertheless, for RDs, challenges arise owing to the rarity of the conditions, complex nomenclatures, and coding or tagging inconsistency between documents or databases. Additionally, author-specific medical terms, abbreviations, and grammatical errors in EHR further hinder biomedical NER. Recurrent NNs proved to be advantageous for automated clinical coding improving representation of RDs in hierarchically-structured medical knowledge [73]. For disease NER, Bhasuran et al. implemented a stacked ensemble approach combined with fuzzy matching using both forward and reverse disease labeling. Additionally, they developed an in-house disease dictionary combining several databases and increasing RD representation. By including the reverse model, they identified RDs with intricate names unidentified by other NER methods [74]. More recently, Xu et al. developed Dic-Att-BiLSTM-CRF (DABLC), a deep attention NN method. By incorporating dictionary-based (using disease ontology) and document-based attention mechanisms, this new method outperformed existing ones at identifying rare and complex disease names [75].

This enhancement of RD coding and tagging, as well as their representation in databases, potentially benefits data/platform interoperability. Indeed, the lack of compatibility between registries hampers the use of patient data for clinical research. With the goal of contributing to the future interconnection between registries, cluster analysis and RF were used to analyze patient registries. Three types of registries with different profiles and informative needs were recognized, namely, public health, clinical and genetic research, and treatment focused [76].

While important advances are occurring in disease NER using AI, models addressing longer sentences are needed to improve complex RDs’ recognition. Nevertheless, this progress is not only optimizing RD annotation and recognition, but also paving the way to platform interoperability.

## 7. AI for CDG

CDG (ORPHA:137) represents a large heterogeneous group of mostly autosomal recessive disorders with impaired synthesis and attachment of glycans to proteins and lipids [77], and defects in the synthesis of glycosylphosphatidylinositol anchors [77,78,79]. Presently, CDG counts over 130 genetic disorders [80], with PMM2-CDG (ORPHA:79318) being the most common N-glycosylation disease [81]. Owing to the fundamental role of glycosylation in a variety of cellular, developmental, and immunological processes, CDG patients often show multiorgan involvement and elevated phenotypic variability [80,82,83,84,85]. As for other RDs, AI has been used to improve research, diagnostics, and therapeutic options search in CDG.

### 7.1. AI for CDG Disease Mechanisms Elucidation

#### 7.1.1. Prediction of Glycosylation Sites

The nature of the chemical linkage between specific acceptor residues in the protein and sugar determines the type of glycosylation; hence, there is a variety of possibilities for the location of glycosylation sites within a protein [82]. The need for more accurate glycosylation sites’ prediction has been pushed by the growing data obtained from genomics and other “omics”. The reliability of this prediction is essential to guide experimental investigations [82]. However, the experimental determination of glycosylation sites in proteins is an expensive and laborious process [82,83]. Hence, a glycosylation prediction program (GPP), using an RF algorithm and pairwise patterns, was created [83]. Putative glycosylation sites in glycoprotein sequences were automatically identified using ensembles of SVM classifiers (Table 1) [82]. Data incorporation from high throughput glycomics and proteomics projects will foster the development of models that can predict the spatial and temporal dynamics of protein glycosylation. This can have a significant impact in basic and clinical research, ultimately improving CDG patients’ quality of life [82,83].

#### 7.1.2. Identification of Golgi Proteins

The Golgi apparatus (GA) is a central hub for membrane trafficking and a fundamental player in post-translation modifications, particularly in glycosylation. Glycosylation is dependent on membrane trafficking for proper localization of its machinery and to bring the substrates for further processing [86,87]. Membrane trafficking at the GA is regulated by several protein complexes, with the conserved oligomeric Golgi (COG) complex being the most important [88]. Defects and/or malfunction of the COG complex are responsible for COG-CDG [88]. Hence, the exact classification of GA proteins may advance diagnosis and basic research, and contribute to targeted drug development in CDG. The identification of the sub-Golgi protein types (isGPT) model using RF and SVM has been developed to accurately identify sub-Golgi protein types, namely trans- and cis-Golgi proteins [89].

### 7.2. AI for CDG Diagnosis, Classification, and Characterization

One of the major pitfalls in CDG is accurate and timely diagnosis [90]. Face2Gene was used to analyze facial photos of a cohort of 31 PMM2-CDG patients. The trained algorithm correctly identified the 31 facial photographs of PMM2-CDG patients and 41 photographs of new patients with a confirmed PMM2-CDG diagnosis. In all cases, PMM2-CDG appeared in the top 10 syndrome matches offered by the tool. A recognizable facial pattern in PMM2-CDG patients was identified and three different age groups (0–5, 6–11, and 12–18) of PMM2-CDG patients were also differentially discriminated [90].

The phenotype-based rare disease auxiliary diagnosis (RDAD) system was used to assist clinicians in the diagnosis of 27 RDs, including PMM2-CDG. RDAD combines four diagnostic models, which successfully ranked the most likely candidate RDs in the top 10 [91]. Thus, these tools could be of added value to PMM2-CDG diagnosis and natural history definition.

Consensus clinical classification is essential for disease characterization and diagnosis. Regarding EXT1-CDG and EXT2-CDG (multiple osteochodromas; ORPHA:321), no taxonomical system was consistent or easy to apply [92]. Using a pioneering ML model (switching NN), 150 variables from 189 patients were analyzed. The resulting classification system followed up disease progression and defined homogenous cohorts of patients, providing insights into disease pathogenesis and helping diagnosis [92]. Strokes and stroke-like episodes (SLEs) are typical manifestations of CDG [93,94]. SLEs are commonly triggered by infections and have been described in about 20–55% of PMM2-CDG patients. The underlying pathomechanisms of SLEs are poorly understood and clinical guidelines are missing [94]. EHRs of 552,898 patients were divided into two age groups (young and old) and analyzed using data augmentation with active-learning selection to predict young stroke [95]. This model was not used in RDs or CDG, but it could be easily transferrable to CDG patients, contributing to better disease management and quality of life.

### 7.3. AI for Therapy Discovery in CDG

Monogenic RDs can be viewed as dosage problems caused by loss- or gain-of-function of a gene, usually altering protein activity. mRNA modeling is a potential strategy to repurpose drugs for these disorders. Connectivity map (https://www.broadinstitute.org/cmap/) and causal reasoning engine were used to search and analyze mRNA “responsiveness” to drugs in 76 RDs, among which were SLC38A9-CDG (ORPHA: 468699), MAN2B1-CDG, and PMM2-CDG [96]. Among the 75 genes × 310 drugs analyzed, 119 different putative drug–gene interactions were identified. Idarubicin showed modest induction of MAN2B1 mRNA expression in fibroblasts [96]. Despite the need for validation of the identified in silico leads in pathophysiologic relevant tissues, these exciting developments have great potential for revolutionizing the CDG therapeutic landscape.

## 8. Discussion and Conclusions

In the “big data” era, there is a growing need to automate tasks that currently require human intervention. In biomedicine, AI technologies have been developed to analyze a diverse array of data, from individual clinical phenotypes in EHR to large and multiparametric patient cohort analysis, attenuating outstanding difficulties in RDs [97].

Medical DSS are computer-based systems that can structure, store, and apply medical knowledge to guide diagnosis and treatment selection. Their application to diagnosis in clinical practice serves three main purposes: (1) to expedite diagnosis; (2) to correct misdiagnosis; and (3) to diagnose previously undiagnosed patients [98,99]. Although various DDSS exist, few use AI in their functioning or methodology. Fewer still focus on RDs, as they pose a bigger challenge owing to phenotypic heterogeneity and information scarcity. Proof of the existence of such gap is the low exome sequencing diagnostic yield in RDs (~25–30%) [29]. AI algorithms performing mutation detection, prediction, and classification can take RDs’ diagnosis to the next level, increasing these figures and uncovering new disease mechanisms and therapeutic targets. Also, As genomic data alone are frequently insufficient to diagnose and characterize RD patients, AI-based multi-omics integrative approaches are being adopted [21,22,23,25,26,28]. Despite these advances, there is room for improvement in AI-mediated diagnosis. For instance, existing splice classifiers normally have insufficient specificity because they focus on local motifs and largely do not account for long-range specificity determinants [29]. Additionally, as multi-omics data are highly heterogeneous, AI strategies performing network-based approaches are required to further promote combinatory analysis of big data [97].

DFs can be distinctive characteristics of RDs, including CDG. Their analysis through image-based DDSS represents a useful and reliable tool [44,51,90]. However, designing and training these models in RDs faces various confounding and detrimental factors, such as small patient cohorts and differences in patient ethnicity. Because a high number of relatively homogeneous images are needed to optimize the model, these limitations may lead to erroneous calculations and/or recommendations [97].

The colossal, dispersed, unstructured, and poorly annotated clinical information presented across medical literature, EHRs, and patient registries worldwide constitutes a greatly untapped resource. Recently developed ML tools to analyze heterogeneous, sparse, and noisy clinical data have shed light on this neglected topic [73,74,75,76,97]. Nevertheless, most advances have occurred in unconcerted actions or in disease niches. Efforts for unified ontologies are needed to implement compatible and interconnected health registries. AI, through NER and NLP exploration, may play a relevant role in this field.

We have listed several examples of how AI has boosted therapeutic development in RDs. These entail the identification of disease biomarkers [70,71,72], the increase of patient recruitment for CTs [67], and the discovery of drugs for repurposing [63]. However, much is still to be done to overturn the low rate of R&D for RDs. AI progress in therapy development has been modest in comparison with that in other areas (e.g., diagnosis). However, we believe that the stimulating results obtained so far associated with the ever-evolving AI frameworks will soon change this scenario. Healthcare, research, and clinical development should be patient-centered [65]. Efforts to increase patient involvement in various fields of healthcare show a growing need to proactively engage with patients [100], and patient organizations have a central role to play [101]. AI technologies can help assess patients’ experience through the analysis of patient reported outcomes and increase patient recruitment and engagement through social media [100,102]. Furthermore, it can be used to monitor patient adherence in CTs [17].

Despite how much AI predictions pitch in to solve RD medical challenges, all results must be experimentally validated to confirm their (bio)medical relevance [17]. Additionally, we would like to emphasize that, for the application of AI in biomedicine, there is no “one size fits all” solution. Biomedical data/issues are complex; AI-based algorithms and methods are numerous and ever-improving. Also, technical and technological limitations must be carefully considered when designing an AI approach in the biomedical context. Our study has some limitations. Firstly, we only searched a Medline database—Pubmed. Although it is the most complete biomedical literature database, insightful data present in other databases may have been lost. Secondly, some of the included manuscripts were not detected in our initial keyword search. This was because of the absence of the keywords related to AI and ML in both the MeSH terms and in the author-defined keywords of the manuscripts.

Healthcare, research, and patient involvement in health decisions are suffering undeniable change. We believe it is important to cease this momentum and further explore AI potentialities to improve RDs patients’ lives. Having said this, many considerations, including ethical and legal issues (e.g., regarding data management, protection, and access equity), need to be carefully investigated to ensure we will build a brighter future.

## Figures and Tables

**Table 1:** List of available artificial intelligence (AI)- and machine learning (ML)-based methods used in rare diseases (RDs) organized by function.

**General Function**	**Specific Function**	**Reference**	**Software/Platform/Algorithm**	**AI/ML Method**	**Disease(s)**
Mutation Detection and Prediction	Predicts the pathogenicity/disease relevance of genetic variants	Alirezaie et al.	CADD https://cadd.gs.washington.edu/	SVM	Several RDs
			ClinPred https://sites.google.com/site/clinpred/	Ensemble classifier (RF and gradient boosting models)	
		Yan et al.	CNVdigest https://github.com/yangxi1016/CNVdigest)	DNorm (conditional random fields, stochastic gradient descent, pairwise learning to rank)	Digeorge syndrome
		Alirezaie et al.	Fathmm-MKL http://fathmm.biocompute.org.uk/fathmmMKL.htm	SVM based on multiple kernel learning	Several RDs
			GenoCanyon http://genocanyon.med.yale.edu/	Unsupervised statistical learning	
			M-CAP http://bejerano.stanford.edu/mcap/	Gradient boosting trees	
			MetaLR	Ensemble classifier	
			Meta-SVM	Meta-analytic SVM	
		Browne et al.	Meta-SNP http://snps.biofold.org/meta-snp/	RF	Mevalonic kinase deficiency
			nsSNP Analyzer http://snpanalyzer.uthsc.edu/	RF	
			PhD-SNP http://snps.biofold.org/phd-snp/phd-snp.html	SVM	
**General Function**	**Specific Function**	**Reference**	**Software/Platform/Algorithm**	**AI/ML Method**	**Disease(s)**
Mutation Detection and Prediction	Predicts the pathogenicity/disease relevance of genetic variants	Browne et al.	PredictSNP http://loschmidt.chemi.muni.cz/predictsnp/	Consensus classifier using the Naïve Bayes classifier, the multinomial logistic regression model, NN, SVM, K-nearest neighbor classifier, and RF	Mevalonic kinase deficiency
		Alirezaie et al.	REVEL https://sites.google.com/site/revelgenomics/	RF	Several RDs
		Buske et al.	SilVA http://compbio.cs.toronto.edu/silva/	RF	Meckel syndrome and other RDs
		Browne et al.	SNAP https://rostlab.org/services/snap2web/	Neural network	Mevalonic kinase deficiency
		Jaganathan et al.	SpliceAI https://github.com/Illumina/SpliceAI	Deep residual NN	RDs with intellectual disability and autism spectrum disorders
		Alirezaie et al.	VAAST Variant Prioritizer (VVP) http://www.yandell-lab.org/software/vaast.html	Probabilistic search ML tool using the CLRT	Several RDs
		Papadimitriou et al.	VarCoPP https://varcopp.ibsquare.be/	RF	Several RDs (including MODY, Kallman syndrome, familial hemophagocytic lymphohistiocytosis, and nontype I cystinuria)
		Carter et al.	VEST https://karchinlab.org/apps/appVest.html	RF	Miller and Freeman Sheldon syndrome
**General Function**	**Specific Function**	**Reference**	**Software/Platform/Algorithm**	**AI/ML Method**	**Disease(s)**
Mutation Detection and Prediction	Predicts the impact of SNVs on protein stability, affinity and functionality	Alirezaie et al.	Eigen http://eigen.tuxfamily.org/	Unsupervised spectral approach	Several RDs
		Browne et al.	I-Mutant http://folding.biofold.org/i-mutant/i-mutant2.0.html	SVM	Mevalonic kinase deficiency
			iStable http://predictor.nchu.edu.tw/istable/	SVM	
			mCSM http://biosig.unimelb.edu.au/mcsm/	Gaussian process regression model	
			MUpro http://mupro.proteomics.ics.uci.edu/	SVM and neural NN	
		Carter et al., Alirezaie et al., Browne et al.	PolyPhen2 http://genetics.bwh.harvard.edu/pph2/	Naïve Bayes classifier	Several RDs’ Mevalonic kinase deficiency
		Browne et al.	PoPMuSiC-2.1/DEZYME http://dezyme.com/	Simple NN	Mevalonic kinase deficiency
	Predicts gene/variant pathogenicity and clinical relevance while integrating phenotypic data	Boudellioua et al.	DeepPVP https://github.com/bio-ontology-research-group/phenomenet-vp	Deep NN	Several RDs
		Bosio et al.	eDiVA http://www.ediva.crg.eu	RF	CF, PKU, and other RDs
		Li et al.	Exomiser https://github.com/exomiser/Exomiser	RF	Several RDs
		Li et al.	Xrare https://web.stanford.edu/~xm24/Xrare/	Gradient boosting decision tree	Several RDs
**General Function**	**Specific Function**	**Reference**	**Software/Platform/Algorithm**	**AI/ML Method**	**Disease(s)**
Decision Support Systems	DDSS based on phenotype	Ronickle et al.	AdaDX https://ada.com/app/	Augmented QMR Bayesian network	Several RDs
		(Basel-Vanegaite et al., Liehr et al., Zarate et al., Marbach et al., Martinez-Monseny et al., Hsieh et al.	Face2Gene https://www.face2gene.com	Deep NN	RDs including Cornelia de Lange syndrome, Emanuel syndrome and Pallister–Killan syndrome, SATB2-associated syndrome
		Rao et al.	HANRD https://web.rniapps.net/gcas/gcas.tar.gz.	Graph convolution-based association scoring	Several RDs
		Jayed et al.	Phen–Gen http://phen-gen.org/	Bayesian network	Several RDs
		Jia et al.	RDAD http://119.3.41.228:8080/RDAD/faq_help.php	Logistic regression, K-nearest neighbor, RF, extra trees, Naïve Bayes, deep NN, and Bayesian averaging algorithm	Several RDs
		Garcelon et al., Garcelon et al.	Dr. Warehouse http://www.drwarehouse.org/	Vector space model	Lowe syndrome, dystrophic epidermolysis bullosa, activated PI3K delta syndrome, Rett syndrome, and Dowling Meara
**General Function**	**Specific Function**	**Reference**	**Software/Platform/Algorithm**	**AI/ML Method**	**Disease(s)**
Disease Classification and Mechanisms’ Elucidation	Data mining for discovery of molecular patterns	Blasco et al., Lagrue et al.	Biosigner https://bioconductor.org/packages/release/bioc/html/biosigner.html	Partial least square discriminant analysis (PLS-DA), RF, and SVM	ALS
	N-, O-, and C-glycosylation sites prediction	Caragea et al.	EnsembleGly https://omictools.com/ensemblegly-tool	Ensembles of SVM	Possible application in human disorders of glycosylation
		Hamby et al.	GPP http://comp.chem.nottingham.ac.uk/glyco/	RF	
	Sub-Golgi proteins identification	Rahman et al.	isGPT http://77.68.43.135:8080/isGPT/	RF and SVM	Possible application in human disorders of glycosylation
	Data clustering	Hoehndorf et al.	FLAME https://github.com/zjroth/flame-clustering/	Fuzzy clustering	Several RDs, including LSDs and Charcot–Marie–Tooth disease 4J
	Disease pathways prediction	Taroni et al.	MultiPLIER https://hub.docker.com/r/jtaroni/multi-plier/ (tag 0.2.0).	Transfer learning	Systemic lupus erythematous, microscopic polyangiitis, and (eosinophilic) granulomatosis with polyangiitis
**General Function**	**Specific Function**	**Reference**	**Software/Platform/Algorithm**	**AI/ML Method**	**Disease(s)**
Disease Classification and Mechanisms’ Elucidation	Prediction models based on gene expression data and anatomical relationships hierarchy	Lee et al.	URSAHD http://ursahd.princeton.edu./jobs/create/	Bayesian network	Refractory anemia with excessive blasts and sideroblastic anemia
	Data mining, clustering, and visualization tools	Dehiya et al.	Weka https://www.cs.waikato.ac.nz/ml/weka/	Collection of ML algorithms	Several RDs (they exemplify for CF and Rett syndrome)

Legend: AI—artificial intelligence; ALS—amyotrophic lateral sclerosis; CADD—combined annotation dependent depletion; CF—cystic fibrosis; CLRT—composite likelihood ratio test; DDSS—diagnosis decision support system; DeepPVP—deep phenomeNET variant predictor; eDIVA—exome disease variant analysis; GPP—glycosylation predictor; HANRD—heterogeneous association network for rare diseases; isGTP—identification of sub-Golgi protein types; LSD–lysosomal storage diseases; M-CAP—mendelian clinically applicable pathogenicity; mCSM—mutation cutoff scanning matrix; ML—machine learning; NER—named entities recognition; NN—neural network; PKU—phenylketonuria; RD—rare diseases; RDAD—rare disease auxiliary diagnosis; RF—random forest; SilVA—silent variant analyzer; SVM—support vector machine; URSAHD—unveiling RNA sample annotation for human diseases; VarCoPP—variant combinations pathogenicity predictor; VEST—variant effect scoring tool; SNP—single nucleotide polymorphism; SNV—single nucleotide variants.

## Supplementary Materials

The following are available online at https://www.mdpi.com/2073-4425/10/12/978/s1, Figure S1: List of all keywords and search combinations used for this literature review, Figure S2: Diagram of the inclusion/elimination process used for manuscript selection, Figure S3: List of keywords used by the script (Python 3.7.3) to create the double and triple search terms presented in Figure S1. This list should be included in an excel file called “keywordsbulk.xls" in order to be used by the script., Table S1: Advantages, disadvantages and some applications of the major AI/ML methods compiled in this review.

## Abbreviations

AI	Artificial intelligence
ALS	Amyotrophic lateral sclerosis
API	Application programming interface
CDG	Congenital disorders of glycosylation
CDWs	Clinical data warehouses
CFML	Characteristic feature mining algorithm
CNVs	Copy number variants
COG	Conserved oligomeric Golgi
CSAX	characterizing systematic anomalies in expression data
CTs	Clinical trials
CVID	Common variable immunodeficiency
DABLC	Dic-Att-BiLSTM-CRF
DeepPVP	Deep phenomeNET variant predictor
(D)DSS	(Diagnosis) decision support systems
eDIVA	Exome disease variant analysis
HER	Electronic health records
GA	Golgi apparatus
GPP	Glycosylation prediction program
HANRD	Heterogeneous association network for rare diseases
HHT	Hereditary hemorrhagic telangiectasia
IBM	Inclusion body myositis
IR	Infrared
isGPT	identification of sub-Golgi protein types
LSDs	Lysossomal storage diseases
ML	Machine learning
MPS II	Mucopolysaccharidosis type II
NER	Named entities recognition
NGS	Next generation sequencing
NLP	Natural language processing
NN	Neural network
PCA	Principal component analysis
PH	Pulmonary hypertension
QMR	Quick Medical Reference
RDAD	Rare disease auxiliary diagnosis system
RDs	Rare diseases
R&D	Research and development
RF	Random forest
SilVA	Silent variant analyzer
SLE	Stroke-like episodes
SNPs	Single nucleotide polymorphisms
SNVs	Single nucleotide variants
SS	Synovial sarcoma
SVM	Support vector machine
URSAHD	Unveiling RNA sample annotation for human diseases
VarCoPP	Variant combinations pathogenicity predictor
VEST	Variant effect scoring tool
WES	Whole-exome sequencing
WGS	Whole genome sequencing
