# Supplementary material for: Artificial Intelligence (AI) in Rare Diseases: Is the Future Brighter?
Source: Genes (Basel). 2019 Nov 27;10(12):978. doi: 10.3390/genes10120978 (PMC6947640; doi:10.3390/genes10120978)
Supplement: Supplementary file 1 [file genes-10-00978-s001.pdf]

Double combination keyword search

- Rare Disease
- Rare Disorder
- Congenital Disorders of Glycosyation
- CDG
- Metabolic disorders

\+

- Artificial intelligence
- Computational intelligence
- Machine intelligence
- Computer reasoning
- Computer assistance learning
- Machine learning
- Deep learning
- Deep neural knowledge
- Big data
- Data mining

Triple combination keyword search

- Rare Disease
- Rare Disorder
- Congenital Disorders of Glycosyation
- CDG
- Metabolic disorders

\+

- Artificial intelligence
- Computational intelligence
- Machine intelligence
- Computer reasoning
- Computer assistance learning
- Machine learning
- Deep learning
- Deep neural knowledge
- Big data
- Data mining

\+

- Diagnosis
- Drug repositioning
- Drug repurposing
- Therapies
- Drug development
- Clinical trials
- Patient recruitment
- Medical data
- Preclinical research
- Clinical development

Figure S1 – List of all keywords and search combinations used for this literature review.

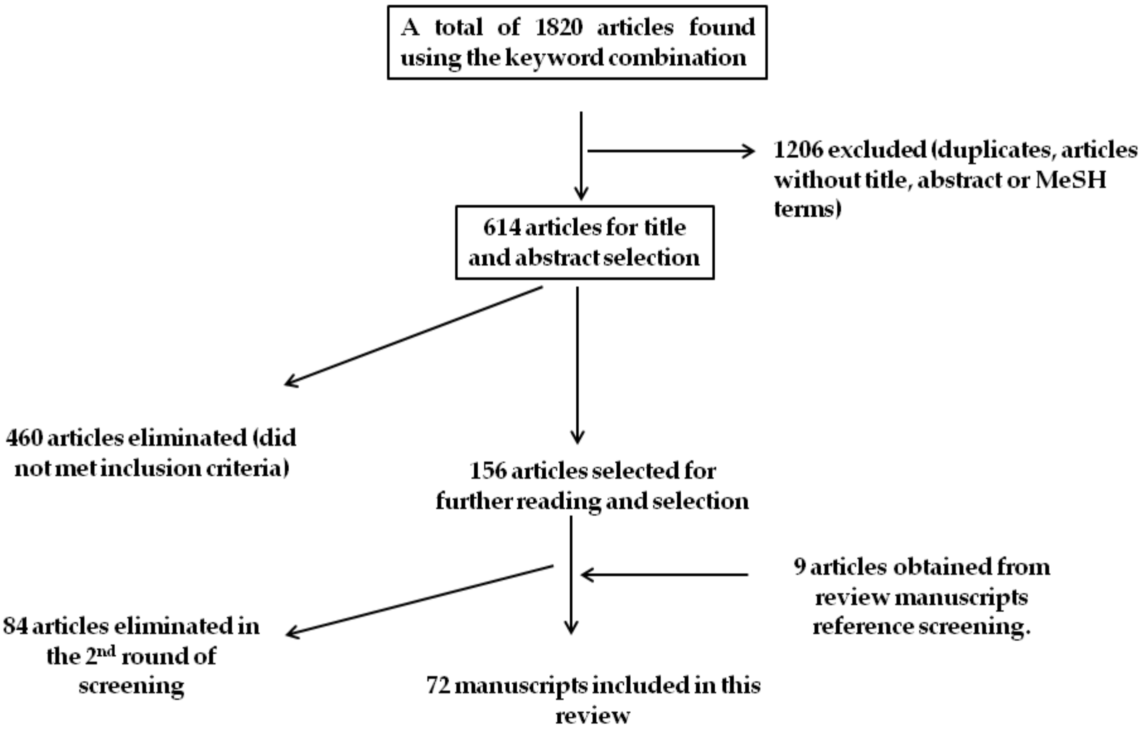

Figure S2 – Diagram of the inclusion/elimination process used for manuscript selection.

Supplementary Note

S1. Python programming language script

The script presented below was used to select the papers and retrieve the correspondent Medline data (Title, Abstract, etc) used for this literature review. This script (Python 3.7.3) was run in a Linux operating system. At line 40 the user should replace "johndoe@mail.com" by his proper email. Furthermore, for the script to work properly, the keywords presented in Fig S3 must be used.

```
import pandas as pd
import numpy as np
import pickle
from Bio import Entrez, Medline

file_loc = "keywordsbulk.xls"
df = pd.read_excel(file_loc, na_values=['NA'],   usecols = "A")
df=df.dropna()
alpha = df['Alpha'].tolist()

df = pd.read_excel(file_loc, na_values=['NA'],   usecols = "B")
df=df.dropna()
beta = df['Beta'].tolist()

df = pd.read_excel(file_loc, na_values=['NA'],   usecols = "C")
df=df.dropna()
gamma = df['Gamma'].tolist()

tripleterms = []
for i in alpha:
    for j in beta:
        for k in gamma:
            aterm = i.lower() + ' ' + j.lower() + ' ' + k.lower()
            tripleterms.append(aterm)

doubleterms = []
for i in alpha:
```

```
    for j in beta:
        aterm = i.lower() + ' ' + j.lower()
        doubleterms.append(aterm)

allterms = tripleterms + doubleterms



Entrez.email = "johndoe@mail.com"
allpmids = []

for ttt in allterms:
    handle = Entrez.esearch(db='pubmed',
                            sort='relevance',
                            retmax='20',
                            term=ttt,
                            usehistory="y")
    pmids = Entrez.read(handle)['IdList']
    allpmids.extend(pmids)

allpmids = list(set(allpmids))

out_handle = open("auxiliary.txt", "w")

allpmidsaux = ','.join(allpmids)
fetch_handle = Entrez.efetch(db='pubmed',
                        rettype='medline',
                        retmode='text',
                       id=allpmidsaux)
data = fetch_handle.read()
fetch_handle.close()
out_handle.write(data)
out_handle.close()

with open("auxiliary.txt") as auxilhandle:
        therecords = Medline.parse(auxilhandle)
        recordslst = list(therecords)

auxilhandle.close()



with open('papers.dat','wb') as filename:
```

```
pickle.dump(recordslst, filename)
```

Alpha	Beta	Gamma
Rare Disease	Artificial Intelligence	Diagnosis
Rare Disorder	Machine learning	Drug repositioning
Congenital Disorders of Glycosylation	Big data	Drug repurposing
CDG	Deep learning	Therapies
Metabolic disorders	Deep neural knowledge	Drug development
	Data mining	Clinical trials
		Patient recruitment
		Medical data
		Preclinical research
		Clinical development

Figure S3 – List of keywords used by the script (Python 3.7.3) to create the double and triple search terms presented in Fig S1. This list should be included in an excel file called "keywordsbulk.xls" in order to be used by the script.

Table S1 – Advantages, disadvantages and some applications of the major AI/ML methods compiled in this review.

General Classification	AI/ML method	Description	Advantages	Disadvantages	Some applications
**Supervised lerning**	SVM	Draws margins between different classes based on the principle of margin calculation. Mainly used for classification [1].	i) Margins are drawn to maximize the distance between the margin and the classes, minimizing the classification error.<br>ii) Can be used for complex data sets with many variables or dimensions [2].	i) Sensitive to noise for small- and medium-sized data sets [3];	i) Biomedical data classification:<br>i.a) Disease classification/subtyping;<br>i.b) Molecular classification/identification (e.g.biomarker identification);<br>ii) Drug discovery (e.g. compound selection/druggability scores);<br>iii) Text categorization.
	Bayesian network	Direct acyclic graph representation of random variables and their conditional probability based on the Bayes' theorem to create decision/belief trees [3].	i) Informs about the interdependency among features<br>ii) Avoids overfitting<br>iii) Robust against missing data [3,4].	i) When there are too many features;<br>ii) The network structure can be difficult to interpret [3].	i) Clustering and classification purposes [1].<br>ii) Disease classification/modeling [4];

Table S1 – Cont.

General Classification	AI/ML method	Description	Advantages	Disadvantages	Some applications
Supervised learning	Naïve Bayes classifier	A type of Bayesian network that assumes independence between variables and that each of them depend on the dependent variable.	i) Does not require a lot of training data<br>ii) ability to process complex queries and high dimensional datasets.<br>iii) highly interpretable<br>iv) not sensitive to irrelevant features or noise [3].	The conditional and independence assumption and oversimplifying relationship among features, hardly represents the real world.	i) Clustering and classification purposes [1].<br>ii) Disease classification/modeling [4];
	RF	Consists of a set of decision trees, each providing classification for input data. The final classification is established by the most voted prediction [5].	i) Can handle large and unbalanced (e.g. when a specific subgroup is underrepresented) datasets;<br>ii) High accuracy in classification, as it generates an internal unbiased estimate of the generalization error;	i) Lack of reproducibility, since the building of the trees is random;<br>ii)Interpretation of the final model and subsequent results may be complex;	i) Biomedical data classification:<br>i.a) Medical imaging and data (e.g. patient registries) analysis;<br>i.b) Gene variants pathogenicity prediction;

Table S1 – Cont.

General Classification	AI/ML method	Description	Advantages	Disadvantages	Some applications
**Supervised learning**	RF		iii) Estimates missing data well;<br>iv) Enables a large number of weak or weakly-correlated classifiers to form a strong classifier;<br>v) Runs fast [6].	iii) Cannot cope well with very small samples [6].	i.c) Disease classification/modelling;<br>i.d) Molecular classification/identification (e.g.biomarker identification);
	Ensembles	Combination of various individual learners/tools to form one composite global model. The model can be homogeneous (use only the same type of tools) or heterogeneous (combination of different tools such as: Naïve Bayes, decision trees, NN, etc. [1,7].	i) Higher accuracy and reliability compared to the individual learners;<br>ii) Reduced probability of over fitting, bias and/or error [7];	i) Significant increase in computational cost [8];<br>ii) Increased system processing time [7].	i) Biomedical data classification:<br>i.a) Medical imaging and data (e.g. patient registries) analysis;<br>i.b) Gene variants pathogenicity prediction;<br>i.c) Disease classification/modelling;

Table S1 – Cont.

General Classification	AI/ML method	Description	Advantages	Disadvantages	Some applications
**Supervised learning**	Gaussian process regression model	A non-parametric regression model that defines a prior beliefs/predictions that can be converted into a posteriori predictions [9,10].	i) Flexible model (e.g. a priori beliefs can be shaped through kernel choosing);<br>ii) Make reliable estimates [9].	i) The uncertainty of the model increases away from the training data (this can be both an advantage and disadvantage). In biomedical data this could mean that this model could predict with high accuracy disease progression for the same patient at different time points, but not for different patients.<br>ii) Computationally expensive [10].	i) Gene variant pathogenicity prediction;<br>ii)Longitudinal studies (e.g. Disease modelling)

Table S1 – Cont.

General Classification	AI/ML method	Description	Advantages	Disadvantages	Some applications
**Unsupervised learning**	Fuzzy clustering	Clustering is the assignment of object groups into clusters (groups). In fuzzy/soft clustering data elements belong to more than one cluster and each element is associated to a set of membership levels [11].	i) Ability to handle large-scale data;<br>ii) Easy detection and handling of noisy data and outliers;<br>iii) Ability to deal with data having different types of variables [12].	i) Difficulty in handling outlier points;<br>ii) Dependence of membership values of other cluster centers;<br>iii) Problems handling high dimensional data sets and large number of prototypes;<br>iv) Possibility of coincident cluster generation [12].	i) Data mining;<br>ii) Pattern/image detection/recognition;<br>iii) Biomedical data classification:<br>iii.a) Disease classification/diagnosis.

Table S1 – Cont.

General Classification	AI/ML method	Description	Advantages	Disadvantages	Some applications
**Supervised/Unsupervised learning**	Neural network (NN)	Derives from the biological concept of neurons. It works in three layers: the input layer (takes in information), the hidden layer (processes the information) and the output layer (calculates the final result) [1].	i) Low classification errors;<br>ii) Low memory requirements;	Low interpretability [3];<br>Difficulty in choosing the network architecture;<br>Dependence of several factors (e.g. initial weight values) for algorithm efficient training;<br>The resulting classifier is a "black box", leading to difficulty in understanding the resulting set of weights [13].	i) Biomedical data classification:<br>i.a) Medical imaging and data (e.g. patient registries) analysis;<br>i.b) Gene variants pathogenicity prediction;<br>i.c) Disease classification/modelling;<br>i.d) Molecular classification/identification (e.g.biomarker identification);<br>ii) Drug design and development [14].

Table S1 – Cont.

General Classification	AI/ML method	Description	Advantages	Disadvantages	Some applications
Supervised/Unsupervised learning	Deep NN	NN with more than one hidden layer [15].	i) Capability of automatically extract, from raw data, the appropriate features for the learning task, thus avoiding feature engineering [16].<br>ii) Yields state of the art results in several learning tasks.	i) High computational cost;<br>ii) Difficulty to scale. [8].	Applies to most of the learning problems such as: i) Image and language understanding;<br>ii) Drug discovery, diagnostics;<br>iii) Discovery of biological processes, etc.
	Graph Convolution-based Association Scoring	A spectral graph convolution algorithm for inferring pairwise associations [17].<br>It is a subcategory of NN.	i) Solves the problem of phenotype-driven rare disease gene prioritization wherein the input is a set of pheno-types from clinical cases and the output a ranked list of possible causal genes [17].	i) Only first order convolutions have been studied for GCAS [17].	Hypothetically several areas of applications, however only phenotype-driven rare disease genes prioritization has been tried [17].

Table S1 – Cont.

General Classification	AI/ML method	Description	Advantages	Disadvantages	Some applications
**Supervised/Unsupervised learning**	Transfer Learning	The improvement of learning in a new task by using the knowledge acquired from a different but related task [18].	i) Highly valuable when there isn't enough labeled data to train the algorithm for a specific task [18,19].	i) Negative transfer: when the knowledge comes from a not enough related task the knowledge transfer can hurt the learning performance [19].	i) Classification tasks;<br>ii) Feature selection;<br>iii) Visual tracking [18,20].

NN – Neural network, RF – Random Forest, SVM – Support Vector Machine

References:

1. Dey, A. Machine Learning Algorithms: A Review. **2016**, *7*, 6.
2. Handelman, G.S.; Kok, H.K.; Chandra, R.V.; Razavi, A.H.; Lee, M.J.; Asadi, H. eDoctor: machine learning and the future of medicine. *J Intern Med* **2018**, *284*, 603–619.
3. Ehsani-Moghaddam, B.; Queenan, J.A.; MacKenzie, J.; Birtwhistle, R.V. Mucopolysaccharidosis type II detection by Naïve Bayes Classifier: An example of patient classification for a rare disease using electronic medical records from the Canadian Primary Care Sentinel Surveillance Network. *PLoS ONE* **2018**, *13*, e0209018.
4. Schaub, N.P.; Alimchandani, M.; Quezado, M.; Kalina, P.; Eberhardt, J.S.; Hughes, M.S.; Beresnev, T.; Hassan, R.; Bartlett, D.L.; Libutti, S.K.; et al. A novel nomogram for peritoneal mesothelioma predicts survival. *Ann Surg Oncol* **2013**, *20*, 555–561.
5. Chen, W.; Wang, Y.; Cao, G.; Chen, G.; Gu, Q. A random forest model based classification scheme for neonatal amplitude-integrated EEG. *BioMed Eng OnLine* **2014**, *13*, S4.
6. Nisbet, R.; Elder, J.; Miner, G. *Handbook of Statistical Analysis and Data Mining Applications*; Nisbet, R., Miner, G., Elder, J., Eds.; Academic Press: Boston, 2009; ISBN 978-0-12-374765-5.
7. Baba, N.M.; Makhtar, M.; Abdullah, S.; Awang, M.K. CURRENT ISSUES IN ENSEMBLE METHODS AND ITS APPLICATIONS. . *Vol.* **2005**, 11.
8. Al-Jarrah, O.Y.; Yoo, P.D.; Muhaidat, S.; Karagiannidis, G.K.; Taha, K. Efficient Machine Learning for Big Data: A Review. *Big Data Research* **2015**, *2*, 87–93.
9. Cheng, L.; Ramchandran, S.; Vatanen, T.; Lietzén, N.; Lahesmaa, R.; Vehtari, A.; Lähdesmäki, H. An additive Gaussian process regression model for interpretable non-parametric analysis of longitudinal data. *Nat Commun* **2019**, *10*, 1798.
10. Shi, J.Q.; Wang, B.; Murray-Smith, R.; Titterington, D.M. Gaussian Process Functional Regression Modeling for Batch Data. *Biometrics* **2007**, *63*, 714–723.
11. Suganya, R.; Shanthi, R. Fuzzy C- Means Algorithm- A Review. **2012**, *2*, 3.
12. Grover, N. A study of various Fuzzy Clustering Algorithms. *IJER* **2014**, *3*, 177–181.
13. *Advanced lectures on machine learning: ML Summer Schools 2003, Canberra, Australia, February 2-14, 2003 [and] Tübingen, Germany, August 4-16, 2003: revised lectures*; Bousquet, O., Luxburg, U. von, Rätsch, G., Eds.; Lecture notes in computer science, Lecture notes in artificial intelligence; Springer: Berlin ; New York, 2004; ISBN 978-3-540-23122-6.
14. Krenek, J.; Kuca, K.; Bartuskova, A.; Krejcar, O.; Maresova, P.; Sobeslav, V. Artificial Neural Networks in Biomedicine Applications. In *Proceedings of the 4th International Conference on Computer Engineering and Networks*; Wong, W.E., Ed.; Springer International Publishing: Cham, 2015; Vol. 355, pp. 133–139 ISBN 978-3-319-11103-2.
15. Lim, J.; Bang, S.; Kim, J.; Park, C.; Cho, J.; Kim, S. Integrative Deep Learning for Identifying Differentially Expressed (DE) Biomarkers. *Computational and Mathematical Methods in Medicine* **2019**, *2019*, 1–10.
16. LeCun, Y.; Bengio, Y.; Hinton, G. Deep learning. *Nature* **2015**, *521*, 436–444.

17. Rao, A.; Vg, S.; Joseph, T.; Kotte, S.; Sivadasan, N.; Srinivasan, R. Phenotype-driven gene prioritization for rare diseases using graph convolution on heterogeneous networks. *BMC Med Genomics* **2018**, *11*, 57.
18. Kaboli, M. A Review of Transfer Learning Algorithms. *[Research Report] Technische Universität München.* **2017**, 68.
19. Torrey L, S.J. *Transfer Learning.*; In E. Soria, J. Martin, R. Magdalena, M. Martinez & A. Serrano, editor, Handbook of Research on Machine Learning Applications. IGI Global.;
20. Ruder, S.; Peters, M.E.; Swayamdipta, S.; Wolf, T. Transfer Learning in Natural Language Processing. In Proceedings of the Proceedings of the 2019 Conference of the North; Association for Computational Linguistics: Minneapolis, Minnesota, 2019; pp. 15–18.
